# Improved functional overview of protein complexes using inferred epistatic relationships

**DOI:** 10.1186/1752-0509-5-80

**Published:** 2011-05-23

**Authors:** Colm Ryan, Derek Greene, Aude Guénolé, Haico van Attikum, Nevan J Krogan, Pádraig Cunningham, Gerard Cagney

**Affiliations:** 1 School of Computer Science and Informatics, University College Dublin, Ireland; 2 Department of Toxicogenetics, Leiden University Medical Center, Leiden, The Netherlands; 3 Department of Cellular and Molecular Pharmacology and California Institute of Quantitative Biosciences, University of California, San Francisco, CA 94158, USA; 4 School of Biomolecular and Biomedical Science, University College Dublin, Ireland

## Abstract

**Background:**

Epistatic Miniarray Profiling(E-MAP) quantifies the net effect on growth rate of disrupting pairs of genes, often producing phenotypes that may be more (negative epistasis) or less (positive epistasis) severe than the phenotype predicted based on single gene disruptions. Epistatic interactions are important for understanding cell biology because they define relationships between individual genes, and between sets of genes involved in biochemical pathways and protein complexes. Each E-MAP screen quantifies the interactions between a logically selected subset of genes (e.g. genes whose products share a common function). Interactions that occur between genes involved in different cellular processes are not as frequently measured, yet these interactions are important for providing an overview of cellular organization.

**Results:**

We introduce a method for combining overlapping E-MAP screens and inferring new interactions between them. We use this method to infer with high confidence 2,240 new strongly epistatic interactions and 34,469 weakly epistatic or neutral interactions. We show that accuracy of the predicted interactions approaches that of replicate experiments and that, like measured interactions, they are enriched for features such as shared biochemical pathways and knockout phenotypes. We constructed an expanded epistasis map for yeast cell protein complexes and show that our new interactions increase the evidence for previously proposed inter-complex connections, and predict many new links. We validated a number of these in the laboratory, including new interactions linking the SWR-C chromatin modifying complex and the nuclear transport apparatus.

**Conclusion:**

Overall, our data support a modular model of yeast cell protein network organization and show how prediction methods can considerably extend the information that can be extracted from overlapping E-MAP screens.

## Background

The representation of genetic interactions as networks emerges from continuing studies aimed at characterizing the functions of individual genes, and anticipates systems biology analyses that focus on dynamic network behavior. An important testing ground for such approaches is the single cell eukaryote *Saccharomyces cerevisiae*, for which a more extensive knowledge of individual gene function has been established than for any other organism, and for which by far the largest set of gene-gene and protein-protein interactions has been assembled [1].

For instance, the publication of the *S. cerevisiae *DNA sequence in 1996[2] allowed a set of yeast strains to be generated that each contained a disruption in a single gene [3]. This, and other strain sets, facilitated a wide range of systematic studies aimed at establishing the function of the genes, e.g. [4-8]. At the same time, a number of genetic [9,10] and biochemical methods [11,12] allowed the mapping of > 30,000 protein-protein interactions [13], that could be represented as a large (~4000 node) undirected graph. Within such networks, proteins often form local densely connected network structures that correspond to stable physically associating heteropolymeric complexes that form *in vivo *(e.g. the ribosome, the proteasome). Complexes are an example of groups of proteins that come together to carry out one or more biochemical tasks, for example synthesis of new proteins by the ribosome. Proteins can also associate in a more transient manner in pathways to carry out a biochemical task, often in sequential rapid enzyme-substrate interactions. Because protein functions in the cell operate over different time scales, in different locations, and in different biochemical contexts, understanding how the cell organizes biological events in terms of protein-protein interaction networks has therefore been a major challenge.

One way to improve our ability to interpret protein networks is to combine protein interaction data with additional data sources [14]. In recent years, a distinct class of interaction data has been mapped on a large scale in yeast cells using Synthetic Genetic Arrays (SGA) technology [15]. Termed "synthetic lethal", these interactions describe the negative (i.e. cell death, or a severe growth defect) effects of disrupting two genes, additional ("synthetic") to the effect of disrupting either gene alone [16]. A synthetic lethal interaction implies a functional relationship between the interacting genes. Notably, they are enriched for genes known to be involved in the same biochemical pathways, including cases where the protein products of the gene are known to physically interact and cases where they interact indirectly. Several authors have exploited the distinct characteristics of physical protein interaction data and genetic interaction data to shed light on the organization of yeast cellular pathways [17]. In 2005, a variant of the SGA method was developed that quantified the synthetic effect [18,19]. Notably, this method (Epistatic Miniarray Profiling- or E-Mapping), detects pairwise gene disruptions that cause the yeast to grow more slowly (negative epistasis) or more rapidly (positive epistasis) than the rate predicted using the individual gene disruptions. E-MAP data and protein interaction data has recently been successfully integrated to give insightful views of cellular organization. In particular, several workers have noted clusters of genetic interactions between functional modules [20-22]. Furthermore, these clusters are often 'monochromatic' - predominantly positive or predominantly negative, an observation that agrees with a predicted model of epistasis in the yeast metabolic network, created using flux balance analysis [23].

Although the E-MAP method can in principle be used to quantify the epistasis effect for all pairwise combinations in a model organism, in practice experimental efforts have to date been carried out on smaller gene sets, typically containing 400-800 genes. It has been shown that there is a greater density of genetic interactions between genes whose products share the same function or location [6,24]. Based on this principle, the gene sets chosen for E-MAP screens are selected in order to maximize the number of epistatic interactions identified, and to provide an overview of a broad biological process (e.g. RNA processing, chromosome biology). Interactions that occur between genes involved in different cellular processes are not as frequently measured, yet these interactions are important for providing an overview of cellular organization. A considerable number (~30 - 160) of genes overlap between different E-MAP sets, raising the possibility that the correlation between related genes could be exploited in order to predict epistasis scores not directly measured in an individual E-MAP. Here we develop such an approach and use it to predict new epistatic interactions that enhance our understanding of the yeast interaction network.

To date the majority of methods for predicting genetic interactions have focused on synthetic lethal interactions, while the problem of predicting quantitative epistatic interactions has received less attention. Techniques for the predicting of synthetic lethal interactions have had some success by mixing heterogeneous biological data [25,26]or by exploiting the topology of the underlying protein interaction network [21,27]. Chipman and Singh [28] used random walks on diverse biological networks to predict synthetic lethality while Qi *et al *[29] have used graph based methods, using only the graph of synthetic lethal interactions.

Recently two papers have addressed the problem of imputing missing values within E-MAPs [30,31]. These papers both used information from 'nearest neighbors' to perform the imputation. These neighbor-based techniques exploit the similarity between the interaction profiles of different genes to predict missing values. The simplest neighbor-based technique is K-nearest neighbors, which works as follows: when a gene has a missing value for a condition, the K genes with the most similar interaction profiles are identified, and their measurements for that interaction are combined using a weighted average. We assessed a number of neighbor-based techniques, and found that they could be used to effectively impute the missing values within an E-MAP. Ulitsky *et al*[31] used a similar approach, but incorporated additional genomic features along with neighbor-based information for the imputation. The incorporation of these additional features resulted in minor increases in imputation accuracy, but the authors noted that the applicability of the method was limited to organisms for which such external data were available. Additionally, Ulitsky *et al *used a logistic regression classifier trained on the same features to predict qualitative interactions (positive, neutral, negative) between gene pairs.

Here we develop a prediction approach for E-MAPs and use it to predict new epistatic interactions that enhance our understanding of the yeast interaction network.

## Results

### A method for predicting quantitative epistatic interactions

**Figure 1:**
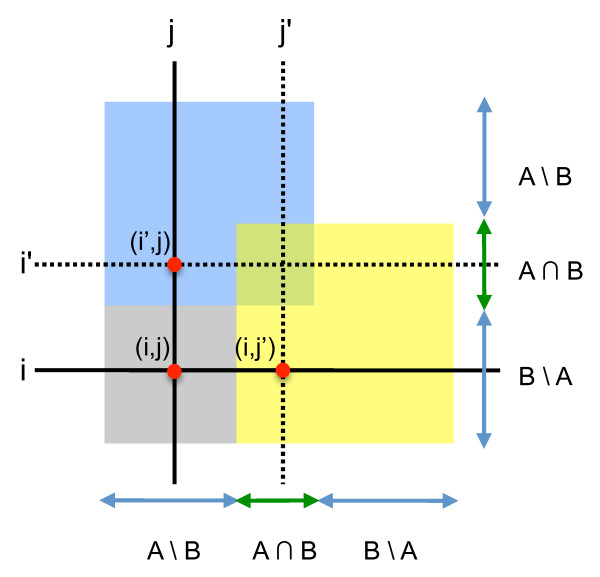
**Symmetric Nearest neighbors**: The area in grey represents the space where we predict interactions. The labels represent the standard set theory definitions - e.g. A\B signifies genes that are in A but not in B. For the missing value *(i, j)*, values from *(i',j) *and *(i,j') *would be combined

To address the problem of inferring epistatic interactions between untested gene pairs, we propose a constrained nearest neighbor-based approach, which exploits the similarity between interaction profiles. Our goal can be most succinctly stated using terms from set theory: given two E-MAPs containing overlapping sets of genes (A and B), we wish to predict scores for interactions between those genes present in A but not in B (A \ B) and those genes present in B but not in A (B \ A) (Figure 1). We achieve this by identifying the nearest neighbors from the genes present in both datasets (A ∩ B). Predictions between genes are only made when one of the genes has a neighbor that is similar enough, i.e. above a specified similarity threshold (using Pearson's correlation as a similarity metric).

The steps of our algorithm are as follows :

1) First we calculated a similarity matrix for the genes within each E-MAP. For each gene in (A \ B), we calculated its similarity to every gene in (A ∩ B). Similarity was defined as the Pearson's correlation coefficient between the interaction profiles of the two genes. The interaction profile for a gene was defined as the vector containing the measured interactions between that gene and all other genes in the E-MAP. Due to missing interactions the correlation is measured over vectors of different sizes - however in 90% of cases there are over two hundred data points in common, and the minimum number of data points used for our experiments was over seventy genes. Even at this minimum number of measurements, the p-value for a correlation of 0.6 is less than 10^-7^. We repeated the process for (B \ A).

2) Using these similarity matrices we identified the nearest neighbor for each gene: the gene with the highest similarity score. If the neighbor was close enough, i.e. above some threshold for similarity, we used it in our imputation.

3) For each interaction pair *(i,j) *where *i *∈ (A \ B) and *j *∈ (B \ A) we checked if *i *had a close neighbor *i'*, and if *j *had a close neighbor *j'*. If *(i,j') *was present in E-MAP B or *(i',j) *was present in E-MAP A, then we used their value as our prediction. If both were available then we used their average as our prediction.

Our method is distinct from existing nearest neighbor imputation methods in three ways: 1) we do not attempt to impute missing or erroneous values, rather we impute values that were never measured in the original screen; 2) we infer the novel interactions by combining pairs of independent but overlapping datasets; and 3) we only provide scores for those interactions which we can estimate accurately.

We carried out our procedure for three published E-MAP studies: Chromosome Biology [32], Signalling [33] and RNA Processing [34] (henceforth referred to as Chromosome, Signalling, and RNA respectively). Within these E-MAPs, the proportion of missing values varies from ~12 - 34% (Table 1). In general, only small sets of overlapping genes are shared between each pair of E-MAPs (e.g. of the 552 genes in the RNA E-MAP, only 125 are also present in the Chromosome E-MAP) yet the correlation between them is high (≥ 0.5, see Table 2) allowing inferences based on shared genes to be exploited by our method. In total, we predicted 34,469 putative epistatic interactions using these overlapping profiles (based on a correlation threshold of 0.6), including 2,240 strongly epistatic interactions (S-score < -2.5 or S-score > 2 [33]), the class of interactions that are most informative in terms of understanding biological function.

**Table 1:** An overview of the E-MAPs analyzed in this study

Dataset	Alleles	% Missing	Reference
Chromosome	754	34.65	[32]
RNA	483	12.69	[34]
Signalling	552	29.53	[33]

**Table 2:** Overlap between different E-MAPs

Dataset A	Dataset B	Common Alleles	Common Interactions	Correlation
Chromosome	RNA	125	4030	0.66
Chromosome	Signalling	142	5321	0.50
RNA	Signalling	63	890	0.60

### Epistasis scores can be accurately predicted by combining datasets

In order to assess the effectiveness of our method, we first performed a 'leave-one-out' style validation procedure using the Chromosome, RNA and Signalling datasets. The values for interactions between genes in (A \ B) and genes in (A ∩ B) are removed one at a time, and an attempt is made to predict them. This is repeated for interactions between genes in (B \ A) and genes in (A ∩ B), allowing us to assess the effect of altering the similarity threshold on the accuracy of the resulting predictions. We used two measures of accuracy: the Pearson's correlation between the predicted and actual interactions, and the normalized root mean squared error (NRMSE, see Eqn. 1). An improvement in accuracy should result in a higher correlation, and a lower NRMSE value.

**Figure 2:**
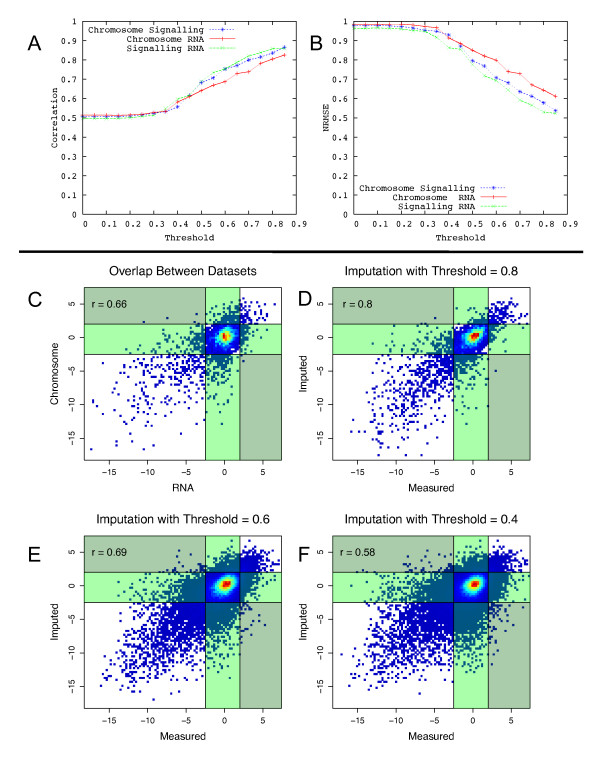
**Similarity threshold vs accuracy**: the impact of the similarity threshold on the accuracy of the predicted S-scores, as measured by correlation between predicted and experimentally observed values **(A) **and NRMSE **(B)**. **(C) **is a density plot showing agreement between independent E-MAP experiments [32,34] and agreement between observed and predicted interactions at thresholds 0.8 **(D)**, 0.6 **(E) **and 0.4 **(F)**. Lines are drawn at the thresholds which have previously been used to identify 'significantly negative' and 'significantly positive' interactions [33]. Interactions in the light green boxes indicate values which should be positive or negative, which have been predicted as neutral(and *vice versa*). Interactions in the dark green boxes indicate values whose polarity has been switched -- significant negatives predicted as positives and *vice versa*.

Both measures show a similar trend (Figure 2). Beyond a minimum similarity threshold of ~0.4, there appears to be an almost linear relationship between the threshold used and the measured accuracy. Scatter plots constructed to compare predicted and experimentally observed epistasis scores show that our predictions have similar variance to independent E-MAP experiments at a correlation threshold of 0.6. Importantly, at a threshold of 0.6 the number of gene pairs misclassified into incorrect epistasis categories (positive classed as negative etc.) is very low (~1%). Thus, for our analysis we used a threshold of 0.6, preferring a smaller number of more accurate predictions to a significantly larger number of less accurate predictions (~35,000 vs ~160,000). However, all predictions made with a threshold of 0.4 and above are given in additional files 1, 2 and 3.

### Predicted epistatic interactions overlap with known interactions and pathways

It has been widely observed that epistatically interacting gene pairs are more likely to share annotated biological properties than randomly selected gene pairs [24]. For instance, gene pairs that show strong epistatic interactions are likely to be involved in the same biological pathways, and so are likely to share Gene Ontology [35] annotations, and to display similar phenotypes. If our predicted epistatic interactions are accurate, then we would expect that they would be similarly enriched for shared annotations and phenotypes. They are also more likely to have been previously identified in genetic interaction screening experiments than randomly selected genes. Furthermore, enrichment for synthetic lethal interactions is likely to be stronger for negative than positive interactions, because synthetic lethal screens primarily report on negative growth phenotypes.

We therefore sought to validate our predictions by comparison with a number of additional data sets. We selected the strongly epistatic pairs from our set of predictions using the thresholds identified in [33] (positive, S-score > 2.0; and negative, S-score < -2.5), and asked whether they are enriched in a variety of annotated properties associated with genetically interacting gene pairs obtained from the literature including the presence of synthetic lethal (genetic) interactions, positive genetic interactions, genes sharing experimental phenotypes, and genes sharing database annotations (Gene Ontology).

Our set of predicted epistatic interactions were indeed enriched for these properties for all three E-MAPs (Table 3).

**Table 3:** Enrichment of predicted interactions between pairs of E-MAPs

Chromosome - RNA						
	**Positive**			**Negative**		

**Dataset**	**Overlap**	**Enrichment**	**p-value**	**Overlap**	**Enrichment**	**p-value**

GO Process	238	1.50	**2.76E-12**	393	1.66	**9.55E-29**
SGD Phenotype	59	2.73	**8.45E-12**	135	4.19	**9.35E-44**
Positive Genetic	20	23.82	**7.29E-21**	3	2.39	0.13
Synthetic Sick	2	1.30	0.67	49	21.35	**1.44E-46**

**Chromosome - Signalling**						

		**Positive**			**Negative**	

**Dataset**	**Overlap**	**Enrichment**	**p-value**	**Overlap**	**Enrichment**	**p-value**

GO Process	56	1.39	**5.98E-03**	121	1.30	**1.12E-03**
SGD Phenotype	10	1.40	2.50E-01	67	4.07	**2.13E-22**
Positive Genetic	3	13.48	**1.56E-03**	2	3.90	0.09
Synthetic Sick	0	0.00	1.00	24	19.98	**3.06E-23**

**RNA - Signalling**						

		**Positive**			**Negative**	

**Dataset**	**Overlap**	**Enrichment**	**p-value**	**Overlap**	**Enrichment**	**p-value**

GO Process	65	2.03	**5.97E-09**	162	2.25	**7.63E-26**
SGD Phenotype	33	5.07	**1.87E-14**	88	6.02	**7.13E-42**
Positive Genetic	16	71.81	**1.19E-24**	2	4.00	0.09
Synthetic Sick	1	2.40	0.34	54	57.77	**1.98E-74**

Enrichment of predicted interactions between the different E-MAPs. Significantly enriched values (*P < 0.01*) are highlighted in **bold**. All p-values in these tables are calculated using Fisher's exact test, and the results are generated using a correlation threshold of 0.6.

As expected, pairs of genes predicted to interact both positively and negatively tend to share GO Process and SGD Phenotype annotations (Table 3), confirming that these genes generally operate within similar biological processes in the cell. Furthermore, the predicted negatively interacting pairs were at least 20-fold more likely to have previously been identified as Synthetic Sick/Lethal in published experiments than random pairs, while pairs predicted to interact positively were at least 13 times as likely to have previously been labeled as such (Table 3). These observations hold for all three pairs of E-MAPs considered, and so are likely to be widely applicable. Overall, validation using both internal (leave-one-out analysis) and external (comparison with annotated biological features) measures supports the assertion that our method successfully generates reliable predictions of epistatic relationships of both positive and negative polarity. The ability of our method to accurately predict the polarity of an predicted epistatic interaction is important, because distinguishing between positive and negative epistasis is critical to mapping the high level relationships between biochemical processes and protein complexes in the cell.

Having used both internal and external procedures to ensure that our predicted interactions were of high quality, we next validated a number of our interactions using a small scale E-MAP in the lab. The results are summarised in Table 4, and the measured interactions are available in additional file 4.

**Table 4:** Accuracy as measured by a new small-scale E-MAP

		Positive		Neutral		Negative	
	**Correlation**	**Precision**	**Recall**	**Precision**	**Recall**	**Precision**	**Recall**

**Predictions**	0.482	0.279	0.182	0.894	0.929	0.491	0.402
**Chromosome**	0.560	0.233	0.306	0.912	0.931	0.566	0.430
**RNA**	0.574	0.358	0.351	0.901	0.914	0.578	0.524
**Kinase**	0.599	0.172	0.458	0.952	0.940	0.528	0.518

The overlap between our predictions and a new small-scale E-MAP. In total 1731 of our predictions were evaluated using this E-MAP. The overlap between the existing E-MAPs and the small-scale E-MAP are given for comparison.

### Improved mapping of epistatic relationships among complexes using inferred interactions

A prime motivation for identifying epistatically interacting genes is to improve our understanding of how the various activities of the cell are coordinated. Kelley and Ideker [21] combined synthetic lethal interactions with physical protein-protein interaction, protein-DNA interaction, and pathway information from the KEGG database [36] to construct 'between pathway' and 'within pathway' models for genetic interactions. This work found that 'between pathway' interactions were the more common class, perhaps because negative interactions typically reflect redundant behavior that is more likely to be found between parallel biochemical pathways than within a single pathway focusing on a given biochemical function. Segre and coworkers [23], used a flux balance model to predict both positive and negative epistatic interactions between gene pairs, and showed that sets of genes involved in coordinated activities tend to show aligned ('monochromatic') epistatic polarity. Bandyopadhyay and coworkers [20] extended these approaches by combining E-MAP data with protein-protein interaction data, to identify modules defined by physical interactions and the largely monochromatic interactions between them.

In order to show how our predicted epistatic interactions can supplement and extend these overviews of the cell, we created a combined E-MAP, consisting of the published RNA, Chromosome and Signalling E-MAPs, augmented with new predictions arising from this manuscript. We mapped the resulting combined E-MAP onto a recently produced high quality list of yeast protein complexes [37], using the method of Bandyopadhyay and coworkers [20,34]. We identified pairs of complexes bridged by genetic interactions which were significantly more negative or positive than one would expect by chance (*P < 0.001*). Additionally we identified complexes whose internal interactions were similarly 'monochromatic'.

**Figure 3:**
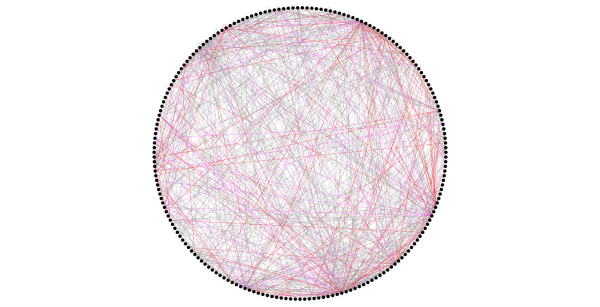
**Novel inter-complex edges generated by newly-inferred epistatic interactions**: Nodes represent protein complexes (as cataloged by Pu *et al *[57]) while edges represent strong net positive or net negative genetic interactions between complexes. Grey edges represent interactions which are unaffected by our predicted interactions, violet edges represent interactions which have been given additional links by our predicted interactions, and red edges represent previously unreported interactions between complexes, established using our method. Edges are only drawn if the median genetic interaction is significantly more positive or negative than one would expect by chance (*P < 0.001*)

Our inferred interactions have two main uses in this context. First, they provide additional evidence for previously proposed connections among protein complexes, and second, they establish new connections. By comparing the resulting network of linked complexes before and after the addition of predicted interactions, we can see which links are a direct result of our predicted interactions. In total 105 'inter complex' links were significantly 'monochromatic' after the addition of our predictions, in other words a set of previously unknown inter-complex links identified with the help of inference. In contrast, the statistical significance of only one 'intra complex' link increased after including our predictions. This apparent discrepancy chiefly arises due to the composition of the E-MAPs published to date, where complexes tend to be represented in only a single E-MAP. Interactions between complexes therefore frequently correspond to links between E-MAPs (Table 5 and Figure 3).

**Table 5:** Inter and Intra complex links

Dataset	Intra Complex Links	Inter Complex Links
Combined without predictions	20	674

Combined with predictions	21	761

Counts of the significant monochromatic epistatic interactions that occur within and between physical complexes defined by Pu *et al*. Inter complex links are genetic interactions that bridge two physical complexes, while intra complex links are genetic interactions within a single complex. Interactions are counted here if they are significantly more negative or positive than one would expect at random (*P < 0.001*).

**Figure 4:**
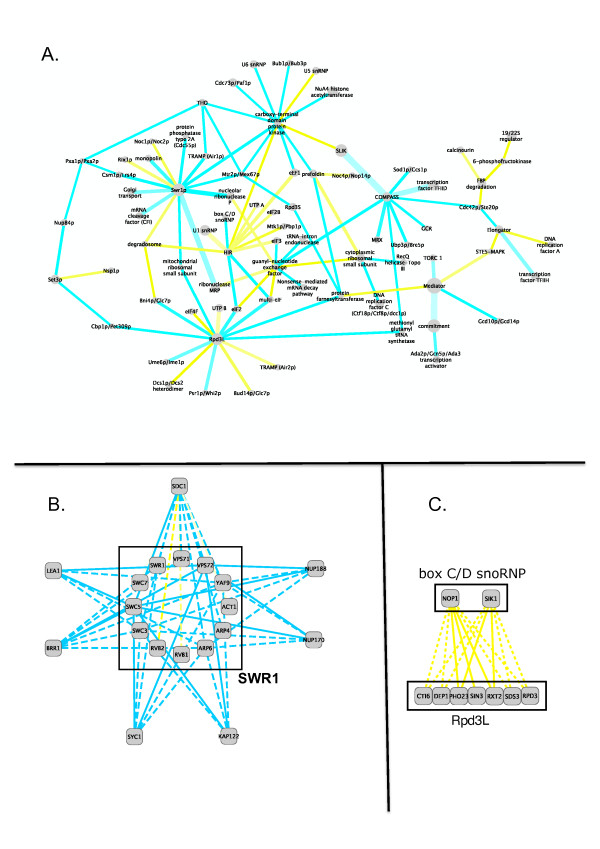
**Novel and Supporting Inter complex edges**: **A**. Monochromatic interactions between complexes whose significance is increased after the addtion of our predicted epistatic interactions. **B**. A close up of the Swr1 complex. **C**. Positive interactions between the "box C/D snoRNP" complex and "Rpd3L" complex

The largest connected component of these 105 novel 'inter-complex' links. Is shown in Figure 4A. This figure is effectively an overview of how cellular processes are organized into modular arrangements, while operating at different hierarchical levels. At the level of the protein complex, individual proteins physically interact, while at the pathway or function level, protein complexes communicate with each other through interactions that are reflected at the epistatic level.

For instance, the cytoplasmic small ribosomal subunit is central to a cluster of protein complexes linking protein translation (guanyl nucleotide exchange factor; prefoldin complex) to DNA biology(e.g. replication factor C, RecQ helicase-Topoisomerase III) and complexes mediating gene expression/chromatin biology (e.g. NuA3 HAT complex). This is consistent with a coordinating role for the ribosome in regulating different aspects of these processes during the cell cycle [38]. Interestingly, the DNA biology gene clusters interact negatively with the ribosome (suggestive of a supportive or co-operative function), while the prefoldin and guanyl nucleotide exchange factor genes interact positively (suggestive of a modifying or regulating relationship).

### Insights into chromatin modifying machines

A good example of how prediction consolidates previously proposed links between complexes, while highlighting new ones, is the SWR-C complex (Figure 4B). This 13 subunit complex is responsible for deposition of the H2A histone variant Htz1 into chromatin in order to promote gene expression and inhibit silencing by heterochromatin [39,40]. Unsurprisingly, SWR-C has been functionally linked to other chromatin modifying complexes including the NuA4 histone acetyltransfersase and the Ino80-C chromatin remodeling complexes [41]. In fact, several proteins are shared among these complexes, including Rvb1p, Arp4p and Yaf9p. Connections between SWR-C and NuA4 or Ino80-C components have previously been observed in E-MAP experiments, and also between SWR-C and another chromatin modifying complex, COMPASS. COMPASS houses a histone H3K4 methyltransferase activity that contributes to gene silencing near telomeres, and is linked to SWR-1 activity via three previously observed E-MAP pairs: SDC1 with SWR1, SWC5 and VPS71. Our predicted interactions however, link the COMPASS subunit SDC1 to eight additional SWR-C subunits: five via negative epistasis (SWC3, VPS72, ARP4, ARP6, YAF9) and three (RVB1, RVB2, ACT1) via weakly positive epistatic interactions (Figure 4 B). We tested the SDC1-SWC5 combination in the small-scale E-MAP (Table 4) and found a very strong negative epistasis (S-score = -15) that agreed with our imputation from the Chromosome Biology/RNA Processing pair of E-MAPs (S-score = -7).

We also predicted an interaction between components of the SWR-C complex (VPS72, SWC5, ARP6, YAF9, SWC3) and the nuclear pore complex (NPC) components Nup170 and Nup188, Figure 4(B). We experimentally tested and validated four of these newly predicted epistatic interactions between these two complexes in the small-scale E-MAP where the predicted S-score agreed with the measured interaction (all S < -3). Interactions between these two components and SWR-C had not been observed before E-MAP screening, but are consistent with observations in the RNA processing E-MAP that connected SWR-C components with the NPC components Nup120p and Mex67p, and a recent report linking Htz1p deposition and the tethering of genes of the nuclear pore [42].

We also experimentally confirmed negative interactions between SWC5 and VPS72 and the nuclear importin KAP122, Figure 4(B), whose gene product binds to the NPC components Nup1p and Nup2p (predicted and observed S-scores = -10/-8 for both interactions). Thus our predicted epistatic interactions suggest new links between SWR-C and components of both the NPC and soluble nuclear transport factors, consistent with a biological rationale whereby histone exchange within nucleosomes is likely to be coordinated with several aspects of the biology of the nucleus [43].

New connections were also made between SWR-C and protein complexes involved in different steps of gene expression. The snRNP Brr1p is involved in snRNP biogenesis pre-mRNA splicing and the cognate gene was predicted to strongly negatively interact with ARP6, VPS72, SWC3, SWC5, and YAF9 Figure 4(B). Two of these interactions were tested and confirmed in the small-scale E-MAP (predicted and observed S-score for SWC5-BRR1 was -8/-9, and for VPS72-BRR1 is -8/-6), while further connections had already been reported in the RNA E-MAP(SWR1-BRR1 and VPS71-BRR1). Similarly, five members of SWR-C were predicted to interact with LEA1, a gene encoding a U2 snRNP component involved in telomere maintenance [44]. Two interactions were tested and confirmed (S-score for VPS72-LEA1 predicated/observed was -7/-7; for SWC5-LEA1 was -7/-8), Figure 4(B). Four members of SWR-C, VPS72, APR6, SWC3 and SWC5, were predicted to negatively interact with the APT cleavage and polyadenylation factor subcomplex component SYC1, with both VPS72 and SWC5 tested and confirmed (predicted and observed S-scores -3/-2 in both cases), Figure 4(B). Thus our predicted interactions link SWR-C to several aspects of gene expression. Interestingly, mRNA splicing and poly(A) cleavage, previously believed to be independent steps of gene expression, are now considered to be linked through large protein complexes that mediate surveillance mechanisms [45].

### Evidence for links between the RPD3L complex and ribosome maturation

Another notable connection is that established between proteins associated with the Box C/D small nucleolar RNAs (snoRNAs) and the Rpd3L histone deacetylase. Genes encoding the Box C/D snRNP associated proteins Nop1 and Sik1 were previously linked to Rpd3L components Pho23, Sin3, and Rtx2, but this was extended in our study to four additional Rpd3L-C genes, Cti6, Dep1, Sds3, Rpd3 (Figure 4C). It is currently unclear how these two complexes functionally interact. The Box C/D snoRNP is responsible for 2'-O-methylation of pre-RNA during ribosome maturation [46], while Rpd3L-C is involved in the regulation of a wide variety of yeast genes [47-49]. Perhaps the extensive genetic interactions between genes encoding the complex subunits, all positive, reflect some communication between ribosome biogenesis and global gene expression changes during growth or during the cell cycle. A number of replication defects that map to the snoRNP component Sik1 (also known as Nop56) have been reported [50], while human snoRNP associated proteins have been co-purified with proteins involved in DNA replication and transcription [51]. Very recently, Rpd3L-C proteins were implicated in replication timing events in yeast [52], so a plausible explanation for epistasis between these complexes could be based on the coordination of DNA replication or the regulation of gene expression or both.

## Discussion

We have developed and implemented a method for predicting E-MAP interactions whose accuracy is similar to that reported for replicate E-MAP screens.

While it is not possible to carry out a direct comparison of our approach to that of previously proposed 'within E-MAP' imputation approaches [30,31], we can gain some insight from a comparison of the reported results.

Ulitsky *et al *evaluated their imputation methods using a number of different models for the source of the missing data. One such model, dubbed 'Cross', was used to model the potential merging of two E-MAP datasets and resembles the problem we are trying to solve. Their method was able to achieve accurate imputations (r > 0.4) in instances when the two E-MAPs shared over 64% of their genes. However, the E-MAPs published to date rarely have an overlap greater than 20% of their genes because they focus on different aspects of cell biology. Additionally, the Ulitsky model was limited to cases where over 50% of the potential interactions were present, a condition not met by any of the overlapping datasets discussed in this paper.

Furthermore, Ulitsky *et al *assessed the accuracy of their imputations within the Chromosome E-MAP by comparing them to the overlap region with the RNA E-MAP. The correlation of their imputed interactions with these measured interactions was ~0.45. Similar to our own results, this was considerably lower than the results expected based on internal cross-validation. This apparent discrepancy can be attributed to the limitations of internal cross validation, and the addition of experiment specific noise.

Finally, we note that the recall for positive interactions is generally lower than that for negative interactions. The authors of both 'within E-MAP' imputation papers have previously highlighted this phenomenon, and put forward plausible explanations for its cause. There are fewer positive interactions overall, and thus fewer examples to draw upon. Also the relative growth rate changes arising from negative interactions are generally higher than those from positive interactions, and these strong negative interactions are likely to be more dominant than positive interactions when assessing the similarity of neighbors.

Overall, comparisons with the published results indicate that we are achieving similar accuracy for our predictions 'between E-MAPs' to that of the reported accuracy 'within E-MAPs'.

One potential caveat to our approach is that we are assuming genes which have similar interaction profiles across a subset of their interactions, i.e. within an E-MAP, will have globally similar interaction profiles. This is potentially not true for all multi-functional genes, for which we could identify neighbors that are locally, but not globally, similar. However given the size of the E-MAPs (hundreds of genes), and the high correlation threshold we set (0.6) it is likely that the number of such spurious neighbor relationships is minimal.

## Conclusion

In summary, we have developed and implemented a procedure for predicting quantitative genetic (or epistatic) interactions using independent experiments that contain overlapping query genes. We show that our predictions are accurate and that the predicted gene pairs share biological properties of experimentally determined gene pairs. We supplemented the known yeast epistasis network (comprising all E-MAP experiments carried out to date) with our new predictions, generating an enlarged yeast epistasis map containing both novel inter-complex links and reinforced links that add confidence to existing links through additional data. Studies using quantitative genetic interactions have increased in number dramatically in recent years. Although we have focused on E-MAP technology, large numbers of interactions continue to be generated using traditional screens or the synthetic genetic analysis(SGA)[53,24] or D-SLAM methods [54]. Furthermore, while they largely originated in yeast models, methods for carrying out epistasis screens have now been developed in other multi-cellular organism models, so it is likely that our method will prove increasingly important in future.

## Materials and methods

### Materials

The three E-MAP datasets analyzed in this paper can be obtained from the supplementary materials of their corresponding papers [32-34].

Genetic interactions can be defined as the divergence(ε) in the observed fitness of strains with two disrupted genes(w_ab_) from the expected fitness. The expected fitness is calculated using the fitness of strains with individual gene disruptions, typically using a multiplicative model(w_a_w_b_).

E-MAPs model this divergence using the S-score, a modified t-score, which takes into account both the magnitude of the divergence, and the variance of the measurements [19]. It thus represents the magnitude of the observed effect and the confidence that it is the result of a true genetic interaction. Each E-MAP consists of a matrix of these S-scores, indicating the type and strength of interaction between each pair of genes under consideration. Negative scores indicate negative epistasis, i.e. the yeast grew more slowly than expected, while positive scores indicate positive epistasis, i.e. more rapid growth was observed. Scores close to zero indicate the probable absence of an interaction between two genes - i.e. they function in independent pathways in the cell.

The GO Slim mapping at the Saccharomyces Genome Database (SGD)[55] was used as the source of gene ontology annotations. These are high-level terms, so annotations which contained more than 1000 genes were filtered out. Phenotype data was also taken from the Saccharomyces Genome Database. Phenotypes associated with more than 175 genes were filtered out, resulting in the removal of terms such as 'inviable', 'viable', and 'haploinsufficient'. Both annotation sets were downloaded on 1^st ^February 2010.

Additionally we investigated whether our predicted interactions were more likely than random to have been previously identified as genetically interacting. For this we used annotations from the Biogrid, version 2.0.61[56]. Our 'positive' test set was comprised of gene pairs annotated with 'Positive Genetic' or 'Synthetic Rescue', while our 'negative' test set was comprised of gene pairs annotated with 'Synthetic Lethality' or 'Synthetic Growth Defect'.

## Methods

### Creating the combined E-MAP

Our combined E-MAP contained all interactions present in the three individual E-MAPs and our predicted interactions. It was created as follows: In cases where the interaction was present in more than one E-MAP, an average of all available interactions was used. In cases where both a measured interaction and a predicted interaction were present, then the measured interaction was used. In cases where there were multiple predictions for a single interaction, the results were averaged.

### Identifying Inter and Intra Complex links

Given the large number of predictions generated, a method to aid their visualization and interpretation was necessary. We exploited the modular architecture of the cell, and focused on interactions at the level of the protein complex. Our set of known protein complexes was taken from a recent manually curated set developed by Pu *et al *[37] as a more up to date alternative to the widely used MIPS dataset. This set consists of 408 complexes backed by evidence from small scale experiments. To identify strong monochromatic genetic interactions between complexes, we used a method developed by Bandyopadhyay *et *al [20]. The median genetic interaction between proteins from two different complexes was compared to the median of *10*^*6 *^equal-sized random samples of genetic interactions. Interactions were considered significant at *P < 0.001*, the same threshold used in [34]. The same method and threshold were used to identify strong genetic interactions within individual complexes.

### Small Scale E-MAP Validation

Validation was performed using a small-scale E-MAP, and is evaluated in Table 4. The double mutant strains were grown and scored using the standard protocols described in [19,18]. We evaluated the performance of our prediction using terms borrowed from the information retrieval community: precision and recall. These are defined as follows:

The small-scale E-MAP was taken to be the 'gold-standard' for identifying strong positive and negative interactions, and precision and recall were evaluated for the overlap between this E-MAP and each of the datasets under consideration in Table 4.

## Authors' contributions

CR, PC, DG and GC designed the study. CR wrote the prediction code. AG performed the laboratory experiments. PC, DG, HvA and NJK contributed reagents/materials/analysis tools. GC and CR performed the analysis of the results. GC, CR, DG and PC wrote the manuscript. All authors read and approved the final manuscript.

## Supplementary Material

Additional file 1 Predicted interactions between the Chromosome and RNA datasets. Click here for file

Additional file 2 Predicted interactions between the Chromosome and Signalling datasets. Click here for file

Additional file 3 Predicted interactions between the RNA and Signalling datasets. Click here for file

Additional file 4 A side by side comparison of the predicted interactions and those measured in a smaller scale E-MAP. Click here for file
